# Lesions to right posterior parietal cortex impair visual depth perception from disparity but not motion cues

**DOI:** 10.1098/rstb.2015.0263

**Published:** 2016-06-19

**Authors:** Aidan P. Murphy, David A. Leopold, Glyn W. Humphreys, Andrew E. Welchman

**Affiliations:** 1 Section on Cognitive Neurophysiology and Imaging, Laboratory of Neuropsychology, National Institute of Mental Health, Bethesda, MD 20838, USA; 2 School of Psychology, University of Birmingham, Edgbaston, Birmingham B15 2TT, UK; 3 Department of Experimental Psychology, Oxford University, Oxford OX1 3UD, UK; 4 Department of Psychology, University of Cambridge, Downing Street, Cambridge CB2 3EB, UK

**Keywords:** depth perception, binocular vision, structure from motion, bistable, parietal cortex

## Abstract

The posterior parietal cortex (PPC) is understood to be active when observers perceive three-dimensional (3D) structure. However, it is not clear how central this activity is in the construction of 3D spatial representations. Here, we examine whether PPC is essential for two aspects of visual depth perception by testing patients with lesions affecting this region. First, we measured subjects' ability to discriminate depth structure in various 3D surfaces and objects using binocular disparity. Patients with lesions to right PPC (*N* = 3) exhibited marked perceptual deficits on these tasks, whereas those with left hemisphere lesions (*N* = 2) were able to reliably discriminate depth as accurately as control subjects. Second, we presented an ambiguous 3D stimulus defined by structure from motion to determine whether PPC lesions influence the rate of bistable perceptual alternations. Patients' percept durations for the 3D stimulus were generally within a normal range, although the two patients with bilateral PPC lesions showed the fastest perceptual alternation rates in our sample. Intermittent stimulus presentation reduced the reversal rate similarly across subjects. Together, the results suggest that PPC plays a causal role in both inferring and maintaining the perception of 3D structure with stereopsis supported primarily by the right hemisphere, but do not lend support to the view that PPC is a critical contributor to bistable perceptual alternations.

This article is part of the themed issue ‘Vision in our three-dimensional world’.

## 1. Introduction

In order to execute appropriate motor responses, such as shaping the hand to grasp an object or navigating through a crowded space, the brain must interpret sensory information to construct an accurate internal representation of the environment. Paramount to human sensorimotor actions is the visual system's ability to infer three-dimensional (3D) depth information from two-dimensional (2D) retinal images. Posterior parietal cortex (PPC) is thought to play a critical role in the transformation of visual information into action-oriented representations, as well as shaping perceptual experience through the selective processing of information [1,2]. The important role of the PPC in perception is most strikingly revealed by the neuropsychological condition of spatial neglect, in which damage to this region, particularly in the right hemisphere, causes deficits of attention and awareness in the contralateral visual hemifield [3–5]. While the 2D mapping of such perceptual deficits onto the visual field reflects the topographic functional organization of parietal cortex [6,7], it is less clear how parietal damage affects perception of 3D space and objects within it.

One important source of visual information for depth perception is binocular disparity: the subtle positional differences between corresponding scene elements in the left and right retinal images, which arise naturally owing to the spatial separation of the eyes. Previous neuropsychological studies suggested that the PPC might play a causal role in the perception of stereoscopic depth from isolated disparity information [8–14]. Specifically, the majority of evidence suggests that regions in the right hemisphere that produce unilateral neglect when damaged (such as PPC) are also necessary for processing depth from disparity in the unaffected hemifield [9,11,15–18], although see [12,19]. However, many of these studies did not have the benefit of accurate anatomical information about the loci of damage, which could therefore only be inferred from the presence or the absence of neglect-like symptoms, and their lateralization. Since lesions to brain regions outside of PPC, including frontal cortex and subcortical structures, are also capable of inducing neglect [20,21] and potentially impairing stereoacuity [22], it has been difficult to link stereopsis specifically to the PPC. Furthermore, despite a wealth of functional neuroimaging evidence for correlations between stereopsis and activity in the intraparietal sulcus (IPS), few imaging studies have reported significant lateralization of these responses, as might be expected from the results in neglect patients [23–26].

Binocular disparity is just one of many visual cues that the brain uses to infer depth information. The inference of depth structure-from-motion (SFM) cues is computationally similar to that from disparity, but exploits motion parallax rather than static positional parallax. Unlike binocular disparity, SFM cues alone are consistent with more than one possible depth arrangement, since the depth order of an object's surfaces remains ambiguous [27]. Under these conditions, perception typically becomes bistable, meaning that an observers' subjective impression of the unchanging stimulus alternates spontaneously between two competing depth interpretations over time. At the single unit level, the perceptual interpretation of these stimuli is reflected in the responses of neurons in cortical visual area V5/MT of macaque monkeys [28,29], and electrical microstimulation of these neurons can induce perceptual biases in the 3D interpretation [30]. Area V5/MT exchanges prominent anatomical connections with PPC [31,32], where many neurons are visually responsive to complex motion features and 3D form [33–35].

The PPC, together with prefrontal areas, has also been implicated in the generation of perceptual alternations during viewing of bistable figures [36–38]. This view is supported by some neuropsychological evidence, which suggests that the rate of spontaneous perceptual alternations during binocular rivalry is reduced in patients with right hemisphere lesions compared with healthy control subjects [39,40]. In addition, transcranial magnetic stimulation (TMS) of PPC, particularly in the right hemisphere, has been shown to influence the rate of perceptual alternations, with perturbation of neighbouring regions of the superior parietal lobule (SPL) and IPS producing somewhat different effects [20,41–43]. However, some of these results appear incompatible, and thus much remains to be learned about how the PPC contributes to perceptual alternations.

This study tested a group of patients with a range of circumscribed and well-characterized unilateral and bilateral parieto-occipital lesions in order to evaluate the causal contribution of PPC to the perception of depth. We tested two aspects of patients' depth perception, the first being the perception of stable stereoscopic depth defined by binocular disparity and the second being the bistable perception of motion-defined depth in an ambiguous SFM stimulus. Using psychophysical methods to test visual sensitivity, we found that stereopsis was severely compromised in patients with lesions to right, but not left, PPC. By contrast, unilateral lesions had little effect on perceptual alternations to ambiguous SFM stimuli or on other bistable stimuli that did not involve depth perception. Patients with bilateral PPC lesions showed, if anything, an increase in perceptual alternation rate compared with controls. Taken together, the results point to a causative role of PPC in the inference of 3D structure, with the perception of stereoscopic depth being strongly lateralized to the right hemisphere.

## 2. Material and methods

### (a) Participants

**Figure 1.**
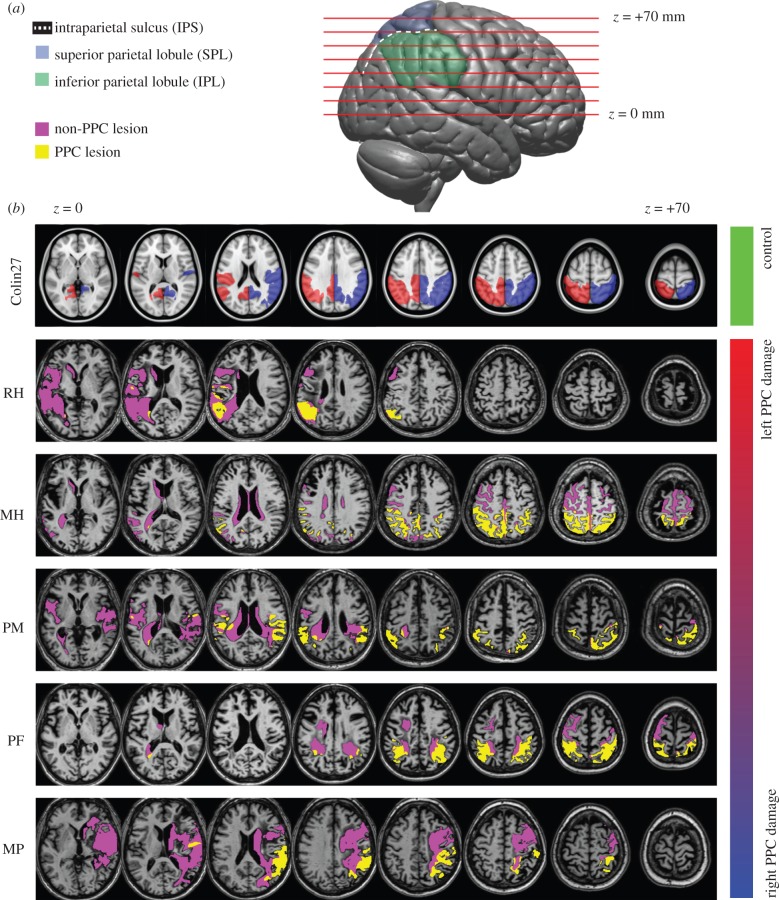
(*a*) Lateral view of the right hemisphere indicating the slice positions shown below and the divisions of the PPC. (*b*) Axial slices of patients' structural MRIs, which were spatially normalized to MNI152 space. For comparison, the top row shows the T1 of a healthy individual (Colin27) also normalized to MNI152 space [45], with left (red) and right (blue) PPC regions of interest (ROIs) overlaid. Patients' lesion masks are overlaid in purple, with lesion voxels within the PPC ROI labelled yellow. In all figures, patients' data are presented in order of PPC lesion lateralization, from left to right (table 2).

Five patients (four males and one female) were recruited from the pool of neuropsychological volunteers established in the Behavioural Brain Sciences Centre at the School of Psychology, University of Birmingham, and had previously participated in the Birmingham Cognitive Screen battery [44]. All patients had acquired brain lesions to parieto-occipital cortex (figure 1), and had been previously evaluated for clinical deficits of spatial neglect and extinction (summarized in table 1). Patients were classed as having a clinical deficit on the basis of whether their test scores were significantly below those of control participants (*n* = 86) with no history of neurological disease (35 males, mean age 67 years, range 47–88 years). Additionally, two healthy age-matched controls (DC and RC, right-handed males, aged 64 and 65) were tested, and 12 younger healthy adults (six males, ages 18–30). All subjects had corrected or normal visual acuity, and none of the patients showed signs of hemianopia based on testing on the Birmingham Cognitive Screen. Data collection from control observers was typically limited to one or two sessions, while patients' data were collected over multiple sessions spanning a total period of 18 months.

**Table 1.** Summary of patient clinical and behavioural details.

initials	sex/age/hand	hand used	years post injury	aetiology	lesion side	extinction^a^ (asymmetry score)	stereo thresholds				SFM	mean percept durations (s)		
							D	F	S-n	C		RS	AM	BR
RH	M/77/L	R	12	stroke	LH	R (+30)	+	+	n.a.	n.a.	+	9.6	n.a.	7.3
MH	M/58/R	R	15	anoxia	LH/B	R (+7)	+	+	+	95%	+	3.6	7.0	3.1
PM	M/69/L	L	>10	stroke	B	L (−2)	−	−	n.a.	77.5%	+	5.1	5.1	n.a.
PF	F/63/R	R	12	stroke	RH/B	L (−12)	−	−	n.a.	70%	+	8.2	6.5	11.0
MP	M/64/L	R	18	aneurism	RH	L (−40)	−	−	−	n.a.	+	8.8	n.a.	8.5

^a^Based on visual extinction test scores, where positive scores indicate right hemifield deficits and negative scores indicate left hemifield deficits [44,46]. AM: apparent motion dot quartet; B, both; BR: binocular rivalry; C, contour; D, dynamic; F, fine; LH, left hemisphere; n.a., not available; RH, right hemisphere; RS: rotating-sphere; S-n, signal-in-noise; SFM: structure-from-motion perception; +, normal; −, impaired.

**Table 2.** Summary of anatomical lesion location and lateralization.

patient	lesion volume whole brain (mm^3^)				lesion volume PPC (mm^3^)			
	total	left	right	Lat. index	total	left	right	Lat. index
RH	105 940	105 940	0	1.00	18 545	18 545	0	1.00
MH	114 857	82 054	35 191	0.70	54 224	36 499	18 841	0.66
PM	116 973	58 679	58 482	0.50	40 863	17 738	23 125	0.43
PF	78 150	42 276	35 884	0.54	42 283	18 812	23 472	0.44
MP	200 596	0	200 596	0.00	30 833	0	30 833	0.00

### (b) Stimuli

Stimuli were programmed in MATLAB (The MathWorks, Natick, MA, USA) with Psychophysics Toolbox extensions [47,48], and presented binocularly in a Wheatstone stereoscope set-up consisting of a pair of ViewSonic P225f CRT monitors (1600 × 1200, 100 Hz) viewed through cold mirrors at a viewing distance of 50 cm. The only exception to this was for the disparity-defined contour task, for which stimuli were presented on a single CRT while participants wore red–green anaglyph glasses. Participants' head position was stabilized through use of a chin rest. For the majority of the bistable and dynamic disparity experiments, eye position was recorded at 1 kHz using an EyeLink 1000 video-based eye tracker (SR Research). Photometric measurements were used to calculate linearized gamma tables (Admesy, Ittervoort, The Netherlands) allowing calibration of the two monitors to produce matched luminance outputs. All stimuli were presented centrally on a mid-grey background, inside a textured border (4° from stimulus edges) consisting of black and white squares (75% density, 0.5° × 0.5°), which served to promote correct vergence posture. In all tasks, participants gave their responses via a configuration of buttons on a gamepad that was customized for each patient such that they could respond easily using the middle and index fingers of their preferred hand.

### (c) Stereoscopic tasks

**Figure 2.**
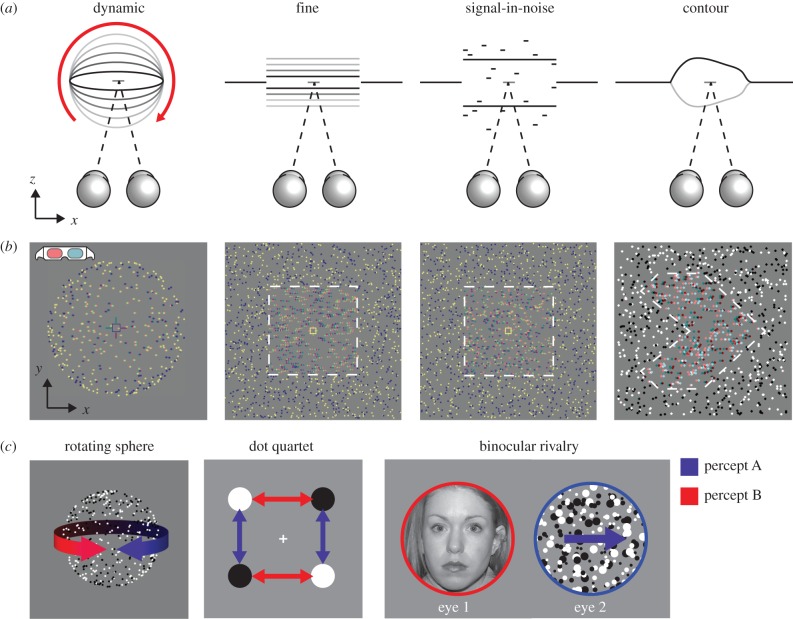
Schematic of stereoscopic and bistable stimuli. (*a*) Schematic view-from-above for the four binocular depth perception tasks presented. (*b*) RDSs are rendered here for red–cyan anaglyph viewing. Dotted outlines illustrate the location of depth edges but were not present in the actual stimuli. (*c*) Schematic illustrations of the three perceptually bistable stimuli: (i) a rotating sphere defined by SFM; (ii) an apparent motion dot quartet; (iii) binocular rivalry between a face and moving dots. For each stimulus, there are two possible perceptual interpretations (indicated here in red and blue), which alternate over time: (i) leftward or rightward rotation; (ii) vertical or horizontal motion; (iii) left or right eye image.

Stereoscopic tasks were performed in blocks consisting of between 8 and 15 repetitions of each stimulus level in a pseudorandom order. All subjects completed one practice block per session, and a minimum of three subsequent blocks in order for the data to be included in the analysis. All random dots stereogram (RDS) stimuli consisted of black and white dots (0.1° radius) on a mid-grey background (figure 2*b*). A fixation marker consisting of dichoptic nonius lines over a binocularly presented square were presented on all tasks except for the disparity-defined contour task, and observers were instructed to maintain fixation during trials.

#### (i) Dynamic stereo task

Random dot kinematograms (RDKs) depicted a rotating sphere (6° diameter), defined by SFM and binocular disparity. The sphere consisted of 400 black and white dots (3 arcmin diameter) distributed randomly across the transparent surface, and rotated about a vertical axis at an angular velocity of 90° s^−1^. Disparity was manipulated parametrically in order to measure psychometric functions, and varied between 0.5 and 14 min of arc between the front surface and the fixation plane. Participants were asked to report which direction (left or right) the front face of the sphere was moving on each trial, and direction was randomized. At smaller binocular disparities, the stimulus becomes bistable, as in the rotating sphere task (see §2d(i) below). On each trial, the stimulus was presented until the observer responded, up to a maximum of 5 s.

#### (ii) Signal-in-noise disparity discrimination

RDSs (8 dots deg^−2^) depicted a central square plane (7 × 7°) at either a crossed or uncrossed horizontal disparity (±6 arcmin) relative to the surrounding border dots (14° × 20°), which lay in the fixation plane. The proportion of dots that appeared at the correct depth in the target plane was parametrically varied between 0 and 100% (seven levels), while the remaining dots were assigned a random depth (±12 arcmin). Stimuli were presented for 500 ms and participants reported whether the central target appeared near or far relative to the fixation plane.

#### (iii) Fine disparity discrimination

RDSs depicted a central square plane inside a border, similar to that presented in the signal-in-noise disparity discrimination task. However, in this case, all dots belonging to the target plane had the same disparity, and this was parametrically manipulated across trials (±0.3, 0.5, 1, 4, 6, 10 and 12 arcmin). Again, stimuli were presented for 500 ms and participants' task was to report whether the central target appeared near or far relative to fixation.

#### (iv) Disparity-defined contours

RDSs depicted convex or concave 3D shapes with symmetrical contours, taken from the 0% noise condition of a previous study by Chandrasekaran *et al*. [49]. Stimuli were scaled to subtend the same visual angle as in the original study (14.4° × 14.4°), and the peak disparity at the centre of each shape was 0.21°. Participants viewed the stimuli through red–green anaglyph glasses and were asked to report the orientation of the axis of symmetry for each stimulus, which was always either horizontal or vertical. Stimuli remained on screen until the participant had given a response. There were 10 stimulus contour shapes, each of which was presented in both vertical and horizontal orientations as convex and concave surfaces, yielding a total of 40 unique stimuli that were presented once each.

### (d) Perceptual bistability tasks

For the bistable experiments, each block lasted 3–5 min, during which observers continuously reported their percept via button press. Data from the first bistable trial of each session were treated as a practice trial and were not included in the data analysis. Fixation markers were presented on 25% of trials, except for the apparent motion task (see below), where the fixation marker was always present. On trials without fixation, observers were instructed to maintain their gaze on the stimuli.

#### (i) Structure from motion

RDKs depicted the orthographic projection of a virtual rotating sphere (6° diameter) with 400 black and white dots (3 arcmin diameter) distributed randomly across the transparent surface, and rotated about a vertical axis at an angular velocity of 90° s^−1^. The apparent direction of rotation was bistable, except during catch periods when binocular disparity was added to disambiguate the direction of rotation. The magnitude of the disparity added during catch periods was set for each observer based on the disparity that enabled a score of 84% correct on the dynamic stereo task (see above). For patients with thresholds outside the tested range of disparities, the maximum disparity was used for catch trials.

#### (ii) Control tasks

In order to determine whether PPC damage influences bistable perception in general, or ambiguous depth perception specifically, we tested subjects on two additional bistable tasks. These tasks both involved motion perception, but neither one elicits the perception of depth.

#### (iii) Apparent motion

The apparent motion dot quartet [50] was composed of two white dots (1° diameter) located in diagonally opposite corners of a rectangular mid-grey background (5° wide × 7–7.2° high), and two black dots of equal size in the other two corners. In the ambiguous condition, the dots switched between these two configurations every 300 ms, with a one frame (10 ms) blank interval interleaved, producing a perception of apparent dot motion that was bistable between horizontal and vertical directions. This frame rate has previously been shown to be well below the threshold for apparent motion perception in patients with parietal damage [51]. Observers fixated a marker located in the centre of the stimulus. During pilot tests, we adjusted the aspect ratio of the stimulus by increasing the vertical distance between dots until observers showed approximately equal probability of reporting horizontal and vertical motion percepts.

#### (iv) Binocular rivalry

Two different images (subtending a visual angle of 8°) were presented to each eye: either a face versus moving dots, or oblique orthogonal drifting gratings (figure 2*c*). Eye of presentation and motion directions were randomized between trials. During catch periods, the contrast of one image was gradually reduced to 20% of its original contrast over a period of 2 s, while the contrast of the other image remained constant. This reduced the probability of the constant image being suppressed and thus increased the probability of it becoming dominant, although it was also possible for observers to perceive the low contrast image.

#### (v) Stabilization

Additionally, the rotating sphere task was performed as described above, but with intermittent presentation of the stimulus in a 1 s on, 1 s off cycle. In healthy observers, this presentation method is known to increase perception durations, i.e. reduce alternation rate [52,53].

### (e) Control task

In the bistable tasks, we randomized the occurrence of ‘catch periods', during which subtle manipulations were applied to the stimuli that temporarily yielded a single objectively correct percept [54,55]. For observers who were capable of discriminating these changes, this ensured that they were attending to the task. For the rotating sphere stimulus, this was achieved by adding small binocular disparities to the sphere, thus yielding an objectively correct direction of rotation. For binocular rivalry, the contrast of one image was gradually reduced over a period of 2 s, and for apparent motion, intermediate frames were added, thus disambiguating the direction of motion. Catch periods were triggered by 15% of button presses that occurred outside of a catch period, and began at a random interval (1–5 s) following the button press. Catch periods lasted 6 s and the disambiguated percept was randomized. During catch periods in the apparent motion task, the direction of movement was disambiguated by briefly presenting an intermediate frame (10 ms), in which the dots appeared halfway between their two normal positions—indicating either horizontal or vertical unambiguous motion. The contrast of these disambiguating dots was reduced (pixel intensity = 5%) to make their presence less obvious. This dot contrast was selected as the lowest contrast at which all observers were able to reliably perceive disambiguated dot motion direction.

### (f) Imaging and analysis

Anatomical MR images were collected at the Birmingham University Imaging Centre using a 3-T Philips Achieva MRI scanner with an eight-channel phased array SENSE head coil. T1-weighted images (1 mm isotropic voxels, TE = 3.8 ms, TR = 8.4 ms) were acquired and processed using the Statistical Parametric Mapping package SPM8 (http://www.fil.ion.ucl.ac.uk/spm) for MATLAB (The MathWorks). Lesion masks were created for each patient, using ITK-SNAP's active contour segmentation [56], and adjusted manually. Patients' structural MR images and lesion masks were then spatially normalized to the MNI152 T1 template using unified segmentation [57,58] and lesion cost function masking [59,60]. Regions of interests (ROIs) for the PPC of each hemisphere were created based on the MNI structural atlas [61,62], and spherical ROIs were created based on published MNI coordinates from previous studies of parietal involvement in perceptual bistability [41–43,63] (see the electronic supplementary material, table S3). Lesion lateralization indices were calculated for the whole brain and PPC ROI by dividing the volume of lesions in the left hemisphere by the volume of lesions in both hemispheres, so that an index of 1 represents lesions exclusively affecting the left hemisphere, while and index of 0 represents lesions exclusively affecting the right hemisphere (table 2). PPC lesion lateralization showed a strong correlation with patients' behavioural performance on a test of visual extinction asymmetry (*R* = 0.99; *p* < 0.01). For each ROI, the proportion of voxels within a 20 mm diameter sphere centred on the MNI coordinate that intersected with the spatially normalized binary lesion mask was calculated. Voxels within the spherical ROI that lay outside of the normalized brain mask were not included.

### (g) Behavioural analysis

For stereoscopic tests, binocular disparity thresholds, sensitivity and confidence intervals were calculated by fitting a cumulative Gaussian psychometric function using a bootstrapping method (Psignifit toolbox; [64,65]), with lapse rate set to 0.01. Binomial maximum-likelihood estimates were calculated using MATLAB's binofit function, which uses the Clopper–Pearson method to calculate confidence intervals [66]. For the disparity-defined contour discrimination task, neither disparity magnitude nor signal intensity was manipulated and, therefore, the proportion of correct responses was analysed.

For perceptual bistability data, percept durations were calculated from observers' active report (via button press). For the rotating sphere and binocular rivalry tasks, percept durations were additionally calculated based on analysis of optokinetic nystagmus (OKN) eye movements, which provide a physiological indicator of perceptual state [55,67,68]. We compared the perceptual time courses extracted from the OKN data to those based on the subjects' perceptual reports (see the electronic supplementary materials). The extracted perceptual time courses were highly correlated with subjective perceptual reports (patient group mean *r* = 0.78 ± 0.04), and transitions in OKN tended to precede reported transitions by approximately 1 s. Inspection of the eye movement data revealed no obvious abnormalities in any of the patients' eye movements in relation to the motion stimuli. All further analyses of perceptual alternations were therefore based on participants' manually reported percepts, as these data were available for all trials. Perceptual dominance periods that were interrupted by catch events or the end of a trial were discarded. Percept durations of less than 300 ms were also discarded as they are likely to be owing to accidental simultaneous button presses.

## 3. Results

### (a) Effects of posterior parietal cortex lesions on stereoscopic depth perception

#### (i) Dynamic stereopsis

**Figure 3.**
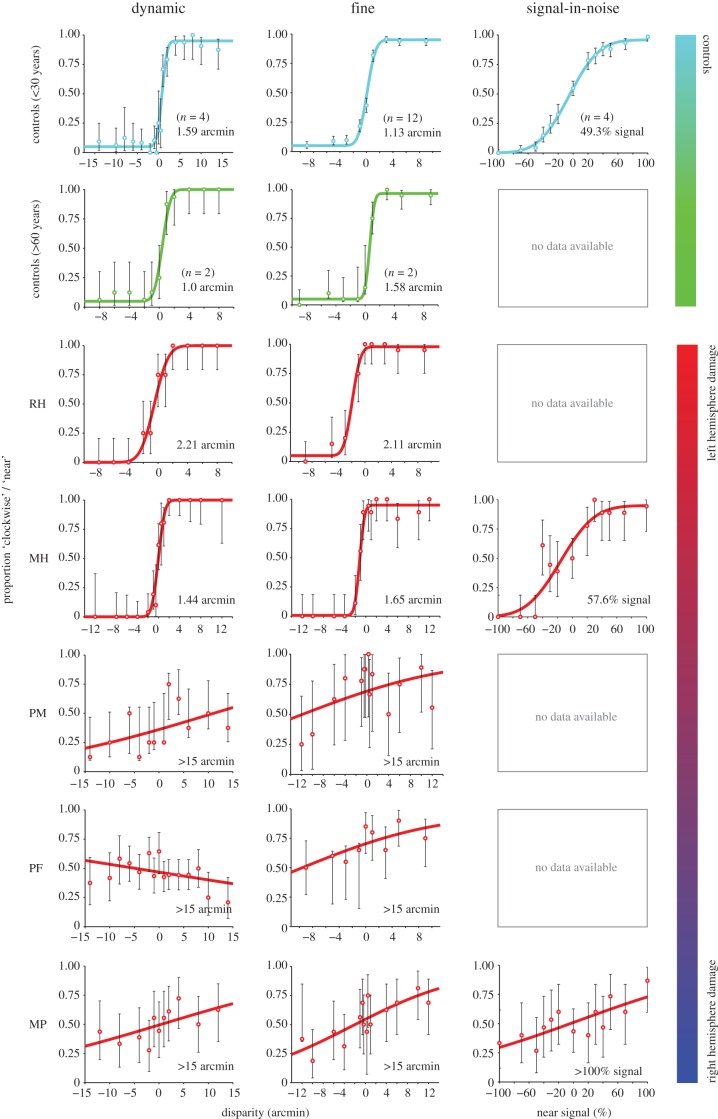
Psychometric functions for all observers on the dynamic, fine, and signal-in-noise binocular disparity tasks. For dynamic disparity (left column), the proportion of ‘clockwise’ responses is plotted as a function of the relative disparity between the front surface of a clockwise rotating sphere and its axis of rotation. For fine disparity (middle column), the proportion of ‘near’ responses are plotted as a function of the disparity of the target plane relative to the border. For signal-in-noise (right column), the proportion of ‘near’ responses is plotted as a function of % signal intensity. Error bars indicate 95% CIs for binomial test. Inset values indicate thresholds at which observers responded correctly to 84% of trials. Data from the young adult (18–30 years) and older adult (60+ years) control groups are indicated in cyan and green, respectively.

In the dynamic stereo experiment, observers viewed a transparent virtual sphere covered in dots, which rotated either clockwise or anti-clockwise about a vertical axis (figure 2*a*,*b* left panels). Observers were instructed to report the direction of motion of the front surface of the sphere (left or right). In the absence of binocular disparity, either the leftward or rightward moving surface could appear in front, and thus the sphere could appear to rotate in either direction. We parametrically manipulated the disparity difference between the front and rear surfaces of the sphere in order to measure observers' sensitivity to dynamic disparity information [30]. We estimated discrimination thresholds by fitting cumulative Gaussian psychometric functions to the data for each subject and establishing the disparity required for subjects to choose the direction consistent with the disparity cue on 84% of the trials (figure 3, left column).

The data revealed a strong effect of PPC lesions on observers' ability to discriminate depth order from dynamic binocular disparity. Three of the patients (PM, PF, MP) performed very poorly on this task and were unable to achieve accuracy above 84% even at the largest disparities tested (15 arcmin). The difficulty did not appear to be related to the capacity to perceive depth *per se*, since prior to the test these same patients reported perceiving a rotating sphere when presented the ambiguous (SFM) stimulus. Although the rotating sphere stimuli were presented for up to 5 s on each trial and participants were not instructed to give speeded responses, most patients took slightly longer to respond on average (across all disparities) compared to control observers.

**Figure 4.**
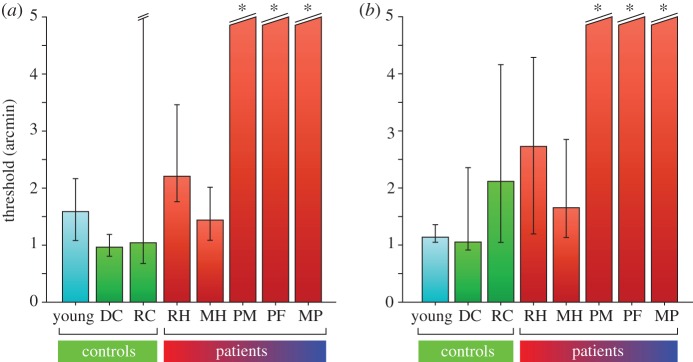
Stereo thresholds for (*a*) dynamic stimuli and (*b*) fine disparity stimuli. Bars show the disparity at which performance reached 84% correct. Error bars show 95% CIs. Asterisk (*): thresholds for patients PM, PF and MP could not be estimated accurately since their performance was less than 84% correct at the largest disparities tested (±14 arcmin).

By contrast, the two other patients (MH and RH) performed the task well, correctly discriminating the direction of rotation even at smaller disparities. Their discrimination thresholds were 1.44 and 2.21 min of arc, respectively, comparable to both the younger control group (*n* = 4, ages 23–28, mean threshold = 1.59 arcmin) and age-matched control subjects DC and RC (0.96 and 1.04 arcmin; figure 4*a*). An important difference between the former and latter groups of patients is likely to be related to a difference in their MRI-determined lesion locations (figure 1 and table 2): those with more extensive damage to the right PPC showed marked impairment in this task, while those with damage restricted primarily to the left PPC did not. We next investigated the generality of these deficits to other tasks involving stereopsis.

#### (ii) Fine stereopsis

We tested the same observers in a fine disparity discrimination task to determine whether the above results were related specifically to the integration of motion and disparity cues or some other aspect of the volumetric stimulus. Observers were presented static random dot stereograms (RDSs) depicting a central square target plane that was either near or far relative to the surrounding surface, which always appeared in the fixation plane (figure 2*a*,*b*, second panels from left). The pattern of discrimination thresholds across observers on this task was similar to that observed in the dynamic stereo experiment (figure 3, middle column and figure 4*b*). The same three patients (PM, PF, MP) were unable to reliably discriminate between near and far conditions in the range of disparities tested (±14 arcmin), even when the stimulus presentation duration was increased to 1 s. However, the slopes of their psychometric functions were greater than zero, which suggests that these observers were able to use some disparity information. By contrast, patients with damage restricted primarily to the left PPC (MH and RH) performed similarly to controls, reliably discriminating surfaces with binocular disparities of only 1.65 and 2.11 arcmin, respectively.

#### (iii) Signal-in-noise stereopsis

We additionally tested patients MH, MP and young adult control observers on the signal-in-noise disparity task. MH's performance, shown in figure 3 (right column), was again comparable to that of young healthy control subjects (*n* = 4), requiring a signal-to-noise ratio (SNR) of 57.6% in order to correctly discriminate depth sign 84% of the time (compared to 49.3% SNR threshold for young controls). By contrast, MP performed poorly even at 100% signal level, as expected from his performance on the fine task.

#### (iv) Disparity-defined contours

We tested patients MH, PM and PF on the disparity contour task (figure 2*a*,*b*, rightmost panels). We compared the results to those of a previous study, in which healthy observers were able to correctly discriminate the axis of symmetry (horizontal or vertical) of the disparity-defined contour with approximately 98% accuracy when the stimuli contained 10% noise and were presented for just 300 ms [49]. In this study, the testing was made easier by removing all noise and by allowing observers unlimited time to view and report their percept. Under these conditions, PM scored 70% correct for convex (uncrossed disparities) and 85% for concave (crossed disparities) shapes, while PF scored 60% and 80%, respectively. Both of these patients had damage to the right PPC. While these responses were statistically above chance (binomial test, *p* < 0.001 and 0.01), they were much less accurate than healthy subjects' performance in the previous study, despite the easier testing conditions. They were also much poorer than a patient with damage primarily to the left PPC (MH), who scored 95% accuracy on this task (two incorrect responses).

### (b) Effects of posterior parietal cortex lesions on spontaneous depth reversals

#### (i) Spontaneous depth reversals

Given the deficits related to perception of depth in moving objects described above, we next asked whether PPC lesions impaired or altered the perception of 3D structure defined exclusively by motion cues (SFM). Specifically, we asked whether the spontaneous depth reversals elicited by an ambiguous rotating sphere (see §2) would be different in patient and control groups. After first establishing that all patients were able to perceive SFM through their verbal descriptions of the rotating sphere stimulus, we presented the ambiguous stimulus in blocks lasting between 3 and 5 min as subjects continuously indicated their perceived direction of motion by pressing one of two buttons.

**Figure 5.**
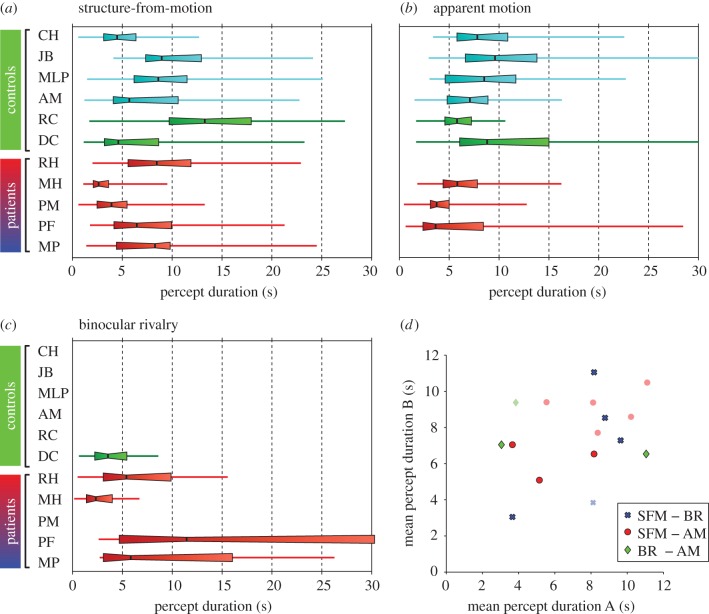
Percept duration distributions for all observers tested on (*a*) the bistable rotating sphere, (*b*) the apparent motion dot quartet and (*c*) the binocular rivalry tasks. Notches represent medians, boxes represent 32nd and 68th percentiles, and error bars represent 5th and 95th percentiles. (*d*) Pairwise comparisons of mean percept durations across the three tasks (*a*–*c*) for each observer.

In contrast to our predictions based on previous studies [39,40], we found that the mean rate of spontaneous depth reversals was similar across groups (figure 5*a*). The range of mean percept durations was comparable to those described elsewhere for healthy observers viewing similar stimuli [42,69], and at the group level, the mean percept durations for patients and controls were not significantly different (Mann–Whitney test, *p* > 0.1). None of the patients with PPC damage showed notable slowing in the rate of perceived reversals of the rotating sphere. In fact, two patients with bilateral PPC damage (MH and PM) were significantly faster in their reversals compared with healthy controls (Mann–Whitney test, *p* < 0.001). These results suggest that a fully intact PPC is not critical for either the perception of 3D SFM or spontaneous depth reversals.

**Figure 6.**
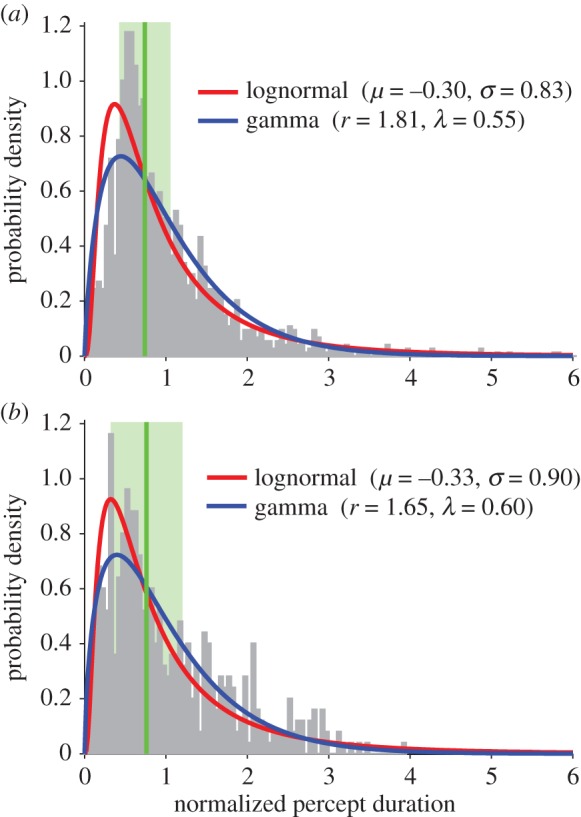
Distribution of normalized percept durations for (*a*) patients (*N* = 5) and (*b*) controls (*N* = 6) for the bistable rotating sphere task. Vertical green lines indicate the distribution medians and the light green bars indicate median absolute deviations.

While mean percept durations provide a simple measure of perceptual stability for comparing observers and groups, they fail to capture more subtle differences in the distribution of percept durations, especially since these distributions are positively skewed. To assess these differences, we fitted probability density functions to each observer's distribution of percept durations (see the electronic supplementary materials) and compared the parameters that described them. Comparing the distribution of shape and scale parameters for the best-fitting lognormal and gamma functions to each observer's percept duration distribution revealed no clear segregation between groups (electronic supplementary material, figure S2), and the parameters for each group were not significantly different from each other (Mann–Whitney test, *p* > 0.1). Finally, since there was no correlation between observers' age and mean percept durations (electronic supplementary material, figure S3), we pooled the normalized percept durations for each group (all controls versus patients) by dividing each percept duration for a given observer by the mean percept duration for that observer (figure 6). Direct statistical comparison of the empirical distributions for the two groups revealed that they were significantly different (Kolmogorov–Smirnov statistic = 0.137, *p* < 0.001). A gamma distribution provided the closest fit to the patient group's data (
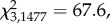
 *p* < 0.001), while the lognormal distribution provided the best fit to the control group's data (
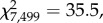
 *p* < 0.001), owing to a greater proportion of short percept durations (normalized value less than 1) in the control group compared with patients.

#### (ii) Perceptual reversals without depth

In order to determine whether PPC damage influences the dynamics of bistable perception more generally, we tested subjects on two additional bistable stimuli that contain motion but do not elicit the perception of depth: the apparent motion dot quartet and binocular rivalry (figure 2*c*). As with the depth reversals on the SFM task, PPC lesions did not appear to disrupt spontaneous perceptual switching for either of the other bistable patterns. For all three patients who were tested on the apparent motion dot quartet task (MH, PM and PF), median percept durations tended to be shorter than those of control observers (figure 5*b*, Mann–Whitney test, *p* = 0.0476). The patients tested on the binocular rivalry task showed a pattern of percept duration distributions that was similar to their performance on the SFM task (figure 5*c*), and the mean percept durations for all three bistable stimuli showed a trend towards being correlated with each other, although this did not reach statistical significance (rotating sphere-binocular rivalry, *r* = 0.61; rotating sphere-dot quartet, *r* = 0.13; figure 5*d*). However, at the group level, the mean percept durations for all patients and controls were not significantly different for any of the bistable tasks, indicating no generalized effect of PPC lesions (Mann–Whitney test, *p* > 0.1).

#### (iii) Experimental controls

In order to control for attentional effects on perceptual alternation, we introduced randomized catch periods in the perceptual bistability experiments, during which the stimuli were temporarily rendered unambiguous. This resulted in only one objectively correct percept during these periods, which observers were expected to report. In the ambiguous rotating sphere experiment, the stimulus during catch periods was identical to that used in the dynamic disparity task, but with a fixed disparity magnitude for each observer. As expected from performance on the dynamic disparity experiment, patients MH and RH responded correctly to 98% (118/120) and 89% (25/28) of catch events, respectively, on the rotating sphere task. Similarly, control observers responded correctly to 93 ± 7% catch periods on average. For the other patients who had shown large thresholds for the dynamic disparity task, the disparity during catch periods was set at 14 arcmin. Surprisingly, two of these patients (MP and PF) were better able to correctly detect the unambiguous direction of rotation from disparity cues in the context of the continuously presented bistability experiment than they had been during the individual trials of the dynamic disparity task (MP = 100%, 4/4; PF = 80%, 12/15; PM = 54% 13/24). Additionally, all observers responded correctly on the majority of catch periods for apparent motion and binocular rivalry stimuli (see the electronic supplementary material, table S1). These results suggest that patients were actively engaged in the perceptual tasks and attending to the stimuli.

We recorded eye position during the majority of the bistable task trials, and presented a central fixation marker on only a small proportion of trials for the rotating sphere and binocular rivalry tasks. When observers were not instructed to fixate, these stimuli naturally elicited OKN eye movements, which provide a physiological indicator of perception [55,67,68]. We compared the perceptual time courses extracted from the OKN data to those based on the subjects' perceptual reports using cross-correlation (electronic supplementary material, figure S1). The extracted perceptual time courses were highly correlated with subjective perceptual reports (patient group mean *r* = 0.78 ± 0.04), and transitions in OKN tended to precede reported transitions by approximately a second (group mean lag = 1.1 ± 0.2 s). Further inspection of the eye movement data revealed no obvious abnormalities in any of the patients' eye movements in relation to the motion stimuli. The eye movement data therefore suggest that observers were actively engaged in the perceptual task and that abnormal eye movements did not influence their perceptual experience.

#### (iv) Perceptual stabilization

**Figure 7.**
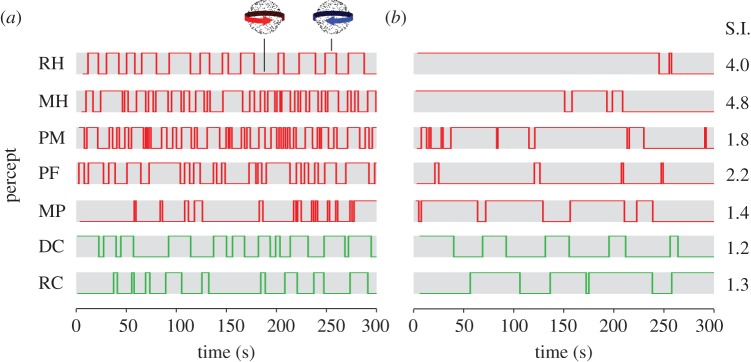
Example data illustrating perceptual stabilization. (*a*) During a 5-min block of continuous presentation of the rotating sphere stimulus, observers reported spontaneous alternations between the two possible percepts (clockwise or anti-clockwise rotation), at a rate that varies between individuals. (*b*) When the same stimulus was presented intermittently (in a 1 s on, 1 s off cycle) the frequency of perceptual alternations was reduced. The right column shows stabilization indices (S.I.) based on all of the data collected for each observer (see the electronic supplementary materials, equation S3 for details).

In addition to measuring alternations in perception during continuous viewing of the bistable stimuli, we tested the effect of intermittent presentation of the same stimuli on observers' perception. In normal observers, intermittent presentation markedly slows down the rate of perceptual switching [52]. Examples of perceptual alternations during 5-min blocks of the rotating sphere task are presented in figure 7, for patients and older control observers. For all observers tested, intermittent presentation of the stimulus reduced the frequency of perceptual alternations compared with continuous viewing. Although the quantity of data available for the intermittent viewing condition was limited owing to the longer percept durations, statistical testing of the available data indicated that the distribution of percept durations during continuous viewing was significantly shorter than those during intermittent viewing (Kolmogorov–Smirnov statistic = 0.204, *p* < 0.001). This reduction in percept durations appeared most pronounced in observers with higher frequencies of alternation during continuous viewing. We quantified the stabilizing effect of intermittent presentation beyond that predicted by the net reduction in stimulus presentation duration by calculating a stabilization index (electronic supplementary material, equation S3). An index value of 1 indicates that the reduction in alternation rate during intermittent viewing is equal to that predicted by the reduced duration of stimulus presentation time, while a larger index indicates a greater stabilization effect. The results indicate that all observers experienced stabilization of perception in the normal range, although this effect was most pronounced in the patients with left hemisphere PPC damage (stabilization indices in figure 7, right column).

## 4. Discussion

We tested visual perception of depth from binocular disparity and motion cues in five patients with lesions to PPC, as well as healthy control observers. We assessed the relationship between lesion locations and behaviour by quantifying lesion lateralization and lesion volumes for specific regions of interest within the PPC that have previously been implicated as playing causal roles in perceptual alternation. The behavioural performance of individual patients appeared to depend on the site of their PPC lesions. In a range of stereo tasks, two patients with damage largely confined to the left hemisphere were able to perceive depth with sensitivities comparable to control subjects, whereas those with right hemisphere PPC damage showed obvious deficits. By contrast, neither right nor left PPC damage significantly diminished the perception of depth SFM. Nor did it disrupt the spontaneous depth reversals normally experienced during continuous viewing of such ambiguous stimuli. In fact, the bistable perception in the two patients with bilateral PPC lesions proceeded at notably faster rate than in the other patients or controls. The following sections discuss, in turn, the role of the PPC in stereopsis, depth from motion and bistable alternations, as well as important caveats surrounding between-subject comparisons of bistable perception.

### (a) Damage to right posterior parietal cortex impairs stereopsis

The two patients (MH and RH) who demonstrated normal ability to discriminate stereoscopic depth were the only two with PPC damage primarily affecting the left hemisphere. This pattern of deficits across subjects is consistent with previous neuropsychological studies suggesting that stereopsis is mediated predominantly by the right hemisphere [9,11,15–17]. The only previous study to report impaired stereopsis following lesions of the left parietal cortex employed the less rigorous ‘Titmus house-fly’ stereotest [12], while another study reported deficits that did not appear to depend on which hemisphere had the lesion [19]. Lateralization of neural responses to binocular disparity has rarely been quantified by functional imaging studies, although examples of greater right hemisphere PPC activation can be found in the literature [24,26,70]. These results go further than previous studies in suggesting specifically that the right PPC is essential for stereopsis, whereas the left PPC is not. Importantly, the patients with right PPC lesions performed comparably to other subjects on visual tasks that did not involve stereopsis (e.g. catch trials during bistable viewing), and performance on the stereo tasks was not associated with the severity of patients' visual extinction but rather with extinction asymmetry. These findings suggest that the observed differences in sensitivity to binocular disparity between patients with left and right PPC lesions were not simply owing to attention. It is noteworthy that patient MH showed both intact local (fine task) and global (signal-in-noise task) stereopsis, despite some degree of damage to right PPC in addition to a more extensive left PPC lesion (figure 1; electronic supplementary material, figure S5). Further consideration of this case may provide an explanation.

MH's injuries are a result of hypoxia caused by carbon monoxide poisoning, which caused thinning of the cortical sheet most prominently in the left parietal lobe but also in the right IPS (rIPS). In that sense, damage to MH's parietal cortex resembles that of another hypoxia patient who, in addition to suffering from visual form agnosia owing to lateral occipital cortex damage, also had damage to left PPC and bilateral atrophy of the IPS [71,72]. That patient, like MH, was found to have preserved depth perception from both static absolute disparities and dynamic relative disparities [73]. This evidence suggests that the type of diffuse damage to PPC and IPS that can result from hypoxic injury may not be sufficient to induce deficits in all aspects of stereopsis. It is worth noting that previous neuropsychological testing revealed that while MH exhibits impaired orientation sensitivity in the left hemifield [74], other functions associated with right PPC remain intact, such as reaching and grasping movements directed to the left hemifield [75–77]. It should be noted that all stereoscopic stimuli were presented centrally in our experiments, and therefore unimpaired stereopsis in just one hemifield could potentially have been sufficient for patients to perform the task.

By contrast, the three patients with prominent lesions to right PPC in this study showed a marked disruption in stereoscopic depth perception, with disparity discrimination thresholds well outside the tested range (more than 14 arcmin) on both the fine and dynamic disparity tasks. This result is consistent with a previous report that patients with right occipito-parietal lesions were unable to detect global stereopsis [18]. Nevertheless, the psychometric fits for data from these observers in this study generally showed positive gradients, indicating some residual sensitivity to disparity information. Similarly, although patients with right PPC damage performed worse than healthy controls on the disparity-defined contour task, their performances were significantly above chance. The only case where a psychometric fit did not show the expected positive slope was for PF in the dynamic stereo task (figure 3), in which SFM provided an additional cue to depth, albeit an ambiguous one. Closer inspection of her responses on this task revealed that she tended to report the same percept across consecutive trials (electronic supplementary material, figure S4)—a response pattern consistent with the idea that PF's perception of the stimulus was based strongly (if not exclusively) on the ambiguous SFM cue, owing to her inability to use the disparity information. The temporal structure of the task (short trials and inter-trial intervals) effectively creates an intermittent presentation schedule, which is known to induce strong perceptual stabilization for bistable SFM stimuli in healthy observers [52], and appears to be preserved in the current patient group.

A variety of factors can contribute to the impairment of stereopsis [78]. One factor that has previously been associated with reduced sensitivity to binocular disparity is age [79–81]. However, in this study patients with left hemisphere lesions and age-matched control subjects performed similarly to, if not better than, younger control subjects, consistent with previous studies that found older adults to perform as well as younger adults on similar stereoscopic tasks [54,73]. This suggests that the deficits observed in the right-hemisphere patients are unlikely to be related simply to age. Another potential cause of impaired stereopsis could be impaired control of eye movements—an action known to involve PPC [82,83]. In particular, vergence eye movements allow registration of the retinal images, which is thought to facilitate the stereo-matching process by minimizing vertical disparities [84]. However, several lines of evidence suggest it is unlikely that vergence deficits could account for the impaired perceptual performance observed in this study. First, PPC disruption primarily affects the latency of vergence eye movements rather than the accuracy [85], and the durations of stimulus presentation used here (1–5 s) were sufficient for delayed vergence movements to be executed well before the stimulus disappeared. Second, even if vergence accuracy was impaired, stereopsis remains robust to vertical disparities of up to 45 arcmin [86]. The available eye movement data suggest that patients exhibited similar patterns of gaze during the dynamic disparity task, irrespective of PPC lesion side (electronic supplementary material, figure S1C). Indeed, disruption of PPC in healthy observers has been shown to impair depth discrimination on a similar signal-in-noise stereoscopic task without affecting vergence eye movements [87]. Interestingly, this disruptive effect of PPC stimulation on stereopsis only occurred when observers had no experience of the stereo task, and was abolished by training the observers on the task. The ability of chronic stroke patients with PPC lesions restricted to the left hemisphere to perceive depth from binocular disparity may therefore reflect long-term recovery of function, which would suggest plasticity and/or redundancy in the way that the brain processes disparity signals. Conversely, lesions to areas outside of PPC could potentially be responsible for the stereo deficits we observed in patients with right PPC lesions, such as lesions to right temporal cortex in patients PM and MP and subcortical damage in patient PF [22]. While a lesion–symptom mapping analysis of a larger patient sample would be required to rule out such possibilities, the right PPC was the primary locus of lesion overlap in these patients and remains the most plausible explanation for the observed deficits in stereopsis.

### (b) Motion perception and structure from motion

All five patients in this study (including those with right occipito-parietal lesions) reported that they were able to perceive the 3D structure of the ambiguous rotating sphere stimulus. This contrasts with the result of a previous study in which five patients with right occipito-parietal lesions reported that they were unable to perceive SFM based on a single trial [18], and another in which patients with parieto-temporal lesions showed impaired perception of motion-defined 2D form [88]. However, unlike these previous studies, our SFM stimuli were not embedded in noise, and thus the 2D object contour was visible even in a single static frame, providing an additional cue to 3D object shape. Similarly, all patients in this study reported being able to perceive apparent motion in a centrally presented dot quartet stimulus. This finding is in agreement with a previous study demonstrating that patients with lesions to right PPC were able to perceive apparent motion in a dot quartet stimulus, provided that the frame rate was below 4 Hz, as was the case for our stimulus [51]. However, evidence that PPC damage causes deficits in dynamic coding for unambiguous dynamic stimuli suggests that PPC may play different roles in the updating of internal representations (ambiguous stimuli) compared to external stimuli [89].

A functionally intact motion-selective area V5/hMT+ is likely a minimum requirement for perception of depth from motion, given the known involvement of this area in processing SFM cues [28,29] and combinations of motion and disparity [30]. This area has previously been shown to be functionally intact in patient MH [90]. However, evidence from neurophysiology suggests that parietal areas (such as LIP in macaques) that receive direct input from MT are also involved in perceptual decisions [91,92], while human neuroimaging also suggests IPS involvement in processing SFM [34,35]. It is therefore of interest that all patients in this study readily perceived SFM. MH and RH's performance on the dynamic stereo task was also comparable to that previously reported for a patient with lateral occipital lesions and three other age-matched controls (aged 53–64) who were tested on a similar task [73]. The reduction in MH's right PPC grey matter therefore appears not to have been sufficient to affect perception of SFM, despite its known involvement in processing other forms of high-level motion [51,93].

Several previous patient studies suggest that both dorsal and ventral visual pathways may be involved in the perception of SFM. A previous study of akinetopsic patient LM revealed that her ability to perceive coherent motion, 2D shape from motion and 3D SFM all broke down with the introduction of moderate levels of noise [94]. Similarly, patients with lesions to ventral visual areas of the right hemisphere were less accurate at discriminating SFM-defined object shapes only when the number of dots defining the object surface was reduced [95]. Thus, ventral visual areas such as lateral occipital cortex seem to be important not only for recognition of 3D object shape from motion and stereo cues [96], but also for extracting such information from noise [87,94]. Further, subtle differences in the location of damage can also effect perception of SFM, since patients with damage to ventral occipital cortex may either show impaired perception of depth from motion with intact motion perception, or vice versa [25,97].

### (c) Spontaneous depth reversals

Previous neuropsychology studies have suggested that lesions to right PPC reduce the rate of perceptual alternations (i.e. increase the duration of perceptual phases) during viewing of bistable stimuli [39,40]. However, in the present bistable perception experiments, none of the patients showed longer percept durations than controls, and the two patients (MH and PM) with bilateral lesions showed significantly *shorter* percept durations than other observers. While it is difficult to estimate the ‘normal’ range of percept durations given the high variability of this measure within the healthy population [42,54], it was notable that these patients showed mean percept durations that were more than 3 s shorter than any of the other observers tested on the rotating sphere stimuli, and that the same two observers also showed the shortest mean percept durations for binocular rivalry and the apparent motion dot quartet, respectively. However, the relationship between the fast perceptual alternation rates and the specific patterns of parietal damage in these patients is not clear.

Previous studies have hypothesized that an antagonistic relationship between posterior and anterior SPL regulates perceptual alternation rate [63,98]. Specifically, this was based on the finding that, in healthy observers, grey matter density in posterior SPL correlates negatively with percept duration, while anterior SPL correlates positively [38,42]. A region-of-interest analysis suggested that the two fast-switching patients have different patterns of bilateral PPC lesions: PM's SPL lesions affect primarily anterior regions whereas MH's lesions affect both posterior and anterior SPL (electronic supplementary material, figure S5). However, for each of the patients who showed fast alternation rates, another patient with a similar pattern of parietal lesions showed a rate of perceptual alternations similar to controls (MP compared to PM, and PF to MH). In contrast to the other patients, RH's parietal lesions are confined to the left inferior parietal lobe, and showed no overlap with any of the SPL ROIs tested. Despite exhibiting severe extinction in his right hemifield, RH showed normal stereopsis and perceptual alternation rates that were not significantly different from control observers.

Two previous studies have examined perceptual alternations during binocular rivalry in patients with right hemisphere lesions [39,40]. In contrast to our results (both for binocular rivalry and SFM), both studies reported that patients with right hemisphere lesions showed a *reduced* frequency of perceptual alternation compared to controls. One of the studies found that this was only the case for patients with unilateral spatial neglect [39], while the other found this effect for all patients with right hemisphere damage, irrespective of neglect symptoms [40]. Since neglect can also result from lesions to areas other than parietal cortex [20], neither study specifically implicates right PPC. However, we found no clear relationship between lesion lateralization and percept durations in our sample, and some evidence to the contrary: one patient whose lesions included limited right PPC damage (MH) exhibited fast alternation rates, while another (PF) showed alternations rates comparable to controls. Despite the apparent right hemisphere dominance for perceptual alternations at the group level emphasized by previous studies [41,63], there appears to be broad individual variation in lateralization [43]. Further, a previous study of a split brain patient showed similar distributions of perceptual dominance durations in each visual hemifield for binocular rivalry [99]. However, there is evidence for hemispheric differences in other parietal-mediated functions that might be related to perceptual selection, such as a dissociation in the contributions of left and right PPC to processing salient stimuli [100].

In addition to PPC, prefrontal cortex (PFC) has also been implicated as playing a causal role in perceptual alternations. Early fMRI studies that contrasted responses to bistable visual stimulation with an unambiguous replay condition reported that fronto-parietal activation was unique to the bistable condition. In addition, neurophysiological evidence suggests that frontal areas show a rapid response that correlates with perceptual transitions and precedes visual responses [101], or correlates with perceptual state independent of response [102,103]. However, several recent fMRI studies suggest that the frontal component of these fronto-parietal activations may primarily reflect the gradual transitions between perceptual states [104], or active reporting of perceptual state [68]. Patients with PFC lesions show similar rates of spontaneous perceptual alternation to healthy controls, although they are less able to voluntarily facilitate perceptual alternations [105], and this finding has been replicated in healthy observers following TMS to PFC [106]. Convergent evidence therefore points towards PPC as playing a dominant role in spontaneous perceptual alternations.

Our observation that intermittent presentation of the ambiguous SFM stimulus prolonged percept durations in patients similarly to controls is further evidence that PPC may not be critical for shaping perceptual dynamics. In healthy observers, brief, repeated presentations of ambiguous stimuli separated by blank intervals of the order of seconds are known to reduce the frequency of spontaneous perceptual alternations [52,53,107]. This phenomenon, referred to as perceptual stabilization, is hypothesized to result from a form of short-term, implicit perceptual memory that promotes the repeated selection of the same percept across consecutive presentations. However, little is known about the neural mechanisms responsible for implementing this process. Some neuroimaging evidence suggests that implicit memory influences perception of bistable stimuli via ‘top–down’ modulation of early visual areas [108–110]; however, several previous studies did not find neural correlates of perceptual memory for bistable motion in area MT [111,112]. The current demonstration of perceptual stabilization in patients with parietal lesions suggests that the putative perceptual memory trace that facilitates this process in not mediated exclusively by PPC, although this does not preclude ‘top–down’ influences [113].

In any experiment where participants are asked to report their subjective perceptual state, an important question is how closely their responses actually reflect their perceptual experience [55]. We used three methods to assess whether observers were actively attending to the bistable stimuli and accurately reporting their percepts (electronic supplementary materials). First, we included random catch periods during which the stimuli were rendered unambiguous, and for which there was only one objectively correct percept [54,55]. Second, we employed ambiguous motion stimuli that elicited specific patterns of reflexive OKN eye movements, and that correlated strongly with perceptual state [55,67,68]. Third, we analysed the distributions of reported percept durations and assessed the goodness of fit with theoretical functions known to provide accurate models for these distributions [114–116]. The results of all three tests suggested that all observers were indeed attending to the tasks and reported their perceptual experience as accurately as possible.

### (d) Conclusion

The results of our study suggest that the ability to perceive depth from binocular disparity in central vision is dependent on the PPC of the right hemisphere. However, the retention of stereopsis in one patient with limited damage to right PPC suggests that there may be some level of redundancy in the processing of binocular disparity information in this area. In contrast to the perception of depth from disparity, all patients tested reported the perception of depth SFM cues. During continuous viewing of a perceptually bistable SFM stimulus, patients with parietal lesions showed variable rates of spontaneous perceptual alternations that were approximately within the normal range, or faster. This stands in contrast to previous reports for patients with putative parietal lesions viewing binocular rivalry stimuli, which found reduced mean alternation rates, although mean rate may not be a statistically reliable measure. Finally, we observed that intermittent presentation of the same ambiguous stimuli induced perceptual stabilization in parietal patients, just as it does in healthy controls. These results suggest a lateralized causal role of the right PPC in the perception of depth from disparity but not from motion, and that the implicit perceptual memory process responsible for stabilizing perception of ambiguous sensory information is not confined to PPC.

## Supplementary Material

Supplementary Materials

## Data accessibility

The data presented in this study are available for download at: http://dx.doi.org/10.5061/dryad.3s06b.
