# Supplementary material for: Lesions to right posterior parietal cortex impair visual depth perception from disparity but not motion cues
Source: Philos Trans R Soc Lond B Biol Sci. 2016 Jun 19;371(1697):20150263. doi: 10.1098/rstb.2015.0263 (PMC4901457; doi:10.1098/rstb.2015.0263)
Supplement: Supplementary Materials [file rstb20150263supp1.pdf]

Supplementary material for Murphy, Leopold, Humphreys & Welchman. Lesions to right posterior parietal cortex impair visual depth perception from disparity but not motion cues, *Phil. Trans. R. Soc. B.,* 10.1098/rstb.2015.0263

Supplementary Materials:

Lesions to right posterior parietal cortex impair visual depth perception from disparity but not motion cues.

Percept duration distributions

Within individuals, the distribution of percept durations is positively skewed, so the mean percept duration (or its reciprocal, alternation rate) is not an ideal metric for perceptual stability. We therefore fitted two functions to the data that have previously been shown to closely approximate the empirical distribution: gamma (1-3), and lognormal (4, 5). Maximum likelihood estimates of shape (k) and scale (λ) parameters for the best lognormal and gamma fits to the empirical percept duration distributions were calculated using the MATLAB functions lognfit and gamfit. The lognormal probability density function (PDF) is given by the equation:

$$f(x|\mu,\sigma) = \frac{1}{x\sigma\sqrt{2\pi}} e^{\frac{-(\ln x-\mu)^2}{2\sigma^2}}$$

(Equation S1)

and the gamma PDF:

$$f(x|k,\lambda) = \frac{1}{\lambda^k \Gamma(k)}\, x^{k-1} e^{-\frac{x}{\lambda}}$$

(Equation S2)

where x = time and Γ(k) = (k-1)!. For gamma rate, shape (k) and scale (λ) parameters for a gamma distribution were fitted to the distribution of switch rates, i.e. the reciprocal of the duration values (1/t) were substituted for *x* (Eq. S2). The result was multiplied by 1/t to obtain the probability, and then divided by the width of a rate bin (Hz) to obtain probability density on a duration axis (seconds).

Goodness of fit for each theoretical distribution to the empirical data was tested using Chi square. The distribution of percept durations for a given individual, and the distribution of mean percept durations across individuals are both positively skewed (4, 6), and were therefore tested using the non-parametric Mann-Whitney and Kolmogorov-Smirnov tests. Finally, to quantify the stabilizing effect of intermittent presentation on bistable perception beyond that predicted by the net reduction in stimulus presentation duration, we calculated a stabilization index (SI):

$$SI = \frac{Continuous\ alternations}{Intermittent\ alternations} \times T_{ON}/(T_{ON} + T_{OFF})$$

(Equation S3)

An SI value of 1 therefore indicates that the reduction in alternations reported during intermittent viewing is equal to that predicted by the reduced duration of the stimulus appearance. Index values above 1 indicate a greater stabilizing effect of intermittent presentation compared to continuous viewing.

Inferring perceptual transitions from eye movements

Eye position was recorded binocularly for the majority of bistable task trials, while a central fixation marker was presented on the remaining trials for the rotating sphere and binocular rivalry tasks. When observers were not instructed to fixate, these stimuli naturally elicited optokinetic nystagmyus (OKN) eye movements, which provide a physiological indicator of perception (7-9). To achieve this, eye position data were first low-pass filtered (25Hz cut-off frequency), and saccades and fast phases of

© The Authors under the terms of the Creative Commons Attribution License http://creativecommons.org/licenses/by/3.0/, which permits unrestricted use, provided the original author and source are credited.

OKN eye movements were then removed by applying a velocity threshold of ±20°s^{-1} in the horizontal direction. The remaining slow phase OKN velocity data were linearly interpolated and low-pass filtered (1Hz), and changes in velocity sign were then identified as perceptual alternations (**Supplementary Figure S1**). Comparison of the extracted versus reported perceptual time courses revealed that extracted perceptual transitions tended to precede reported transitions. Cross-correlations showed that the maximum correlation coefficients (patient group mean r = 0.78 ± 0.04) occurred at a mean lag time of 1.10 ± 0.21s. Inspection of the eye movement data revealed no obvious abnormalities in any of the patients' eye movements in relation to the motion stimuli. The eye movement data therefore suggest that observers were actively engaged in the perceptual task and that abnormal eye movements did not influence their perceptual experience. All further analyses of perceptual alternations were therefore based on participants' manually reported percepts, since these data were available for all trials.

Anatomical description of lesion locations

Patient RH suffered a left-hemisphere stroke that affected the inferior parietal lobe, superior temporal lobe and angular gyrus. Behaviourally, RH exhibits severe extinction of visual events in the right hemifield during bilateral simultaneous stimulation and made left-sided line bisections (10-12). Consistent with the extensive damage to his left hemisphere, RH also shows deep dysphasia, dyslexia and dysgraphia. Patient MH's lesions are the result of an anoxic accident (carbon monoxide intoxication). MRI reveals diffuse and widespread bilateral sulcal widening that is most pronounced in the left parietal lobe, including the occipito-parietal junction, intraparietal sulcus and angular and supramarginal gyri. Subcortically, MH displays ventricular widening and bilateral lesions of the lentiform nuclei and claustrum. Consistent with the left parietal focus of his lesions, MH exhibits unilateral spatial attention bias with extinction in the right hemifield (11). MH also exhibits optic ataxia, but only when reaching to targets in the right visual hemifield with his right arm (11, 13, 14). Functional MRI has previously revealed that MH's motion-selective cortical area V5/hMT+ is intact and responsive (15). Patient PM shows right superior temporal, left angular and supramarginal lesions. There is also a small lesion in the left postcentral gyrus, bilateral putamen and caudate. Patient PF has bilateral lesions to the superior and inferior parietal gyri. Specifically, the left hemisphere lesion extends into the left angular gyrus and the right hemisphere lesion extends slightly into the right postcentral gyrus. There are small lesions in both the left and right putamen which extend into the caudate and another small lesion in the left thalamus. PF exhibits right hemifield extinction and dysgraphia (16). Patient MP has lesions to the right hemisphere affecting the inferior frontal gyrus, the superior temporal gyrus, the supramarginal and angular gyri and the post-central gyrus. The damage caused mild hemiparesis of his previously dominant left arm, although he retained normal sensation. Perceptually, he shows extinction during bilateral simultaneous stimulation (10, 11). Further details regarding the performance of these patients on a range of behavioural tasks are available from previous studies (10, 12-19).

Relationship between perceptual alternations and lesion locations

Previous evidence suggests that specific loci within PPC causally influence perceptual dynamics for bistable stimuli. In order to quantify the extent to which different regions of PPC are affected by lesions in each patient, we used spherical regions of interest (ROIs) centered on previously published MNI coordinates for anterior and posterior superior parietal areas that appear to influence perceptual alternation rates (**Supplementary Table S3**). We calculated the proportion of voxels in each ROI that overlapped with each patient's lesion mask (**Supplementary Table S4, Supplementary Figure S5**), in order to provide a coarse metric of local tissue damage. Plotting these values against mean percept durations did not reveal any clear correlation based on the small patient sample tested.

© The Authors under the terms of the Creative Commons Attribution License http://creativecommons.org/licenses/by/3.0/, which permits unrestricted use, provided the original author and source are credited.

A specific hypothesis postulated by previous studies conducted in healthy observers is that anterior and posterior SPL exert opposing influences on mean percept duration, with disruption of aSPL reducing it and disruption of pSPL increasing it (17, 20). For the ROIs based on these studies, patients MP and PM showed slightly more damage to anterior ROIs than posterior ones (**Supplementary Table S4**). In agreement with the hypothesis, one of these patients (PM) showed shorter mean percept durations on both the rotating sphere and apparent motion dot quartet stimuli. Patient MP's mean percept duration was in the normal range, although there were limited data available for this observer (number of percept durations on the rotating sphere = 17), resulting in a larger standard error. In contrast, patients MH and PF's lesions overlapped both anterior and posterior ROIs, for which the predicted outcome is unclear. MH showed shorter percept durations than average, while PF's were variable across stimuli (close to average for the rotating sphere but shorter than average for apparent motion).

© The Authors under the terms of the Creative Commons Attribution License http://creativecommons.org/licenses/by/3.0/, which permits unrestricted use, provided the original author and source are credited.

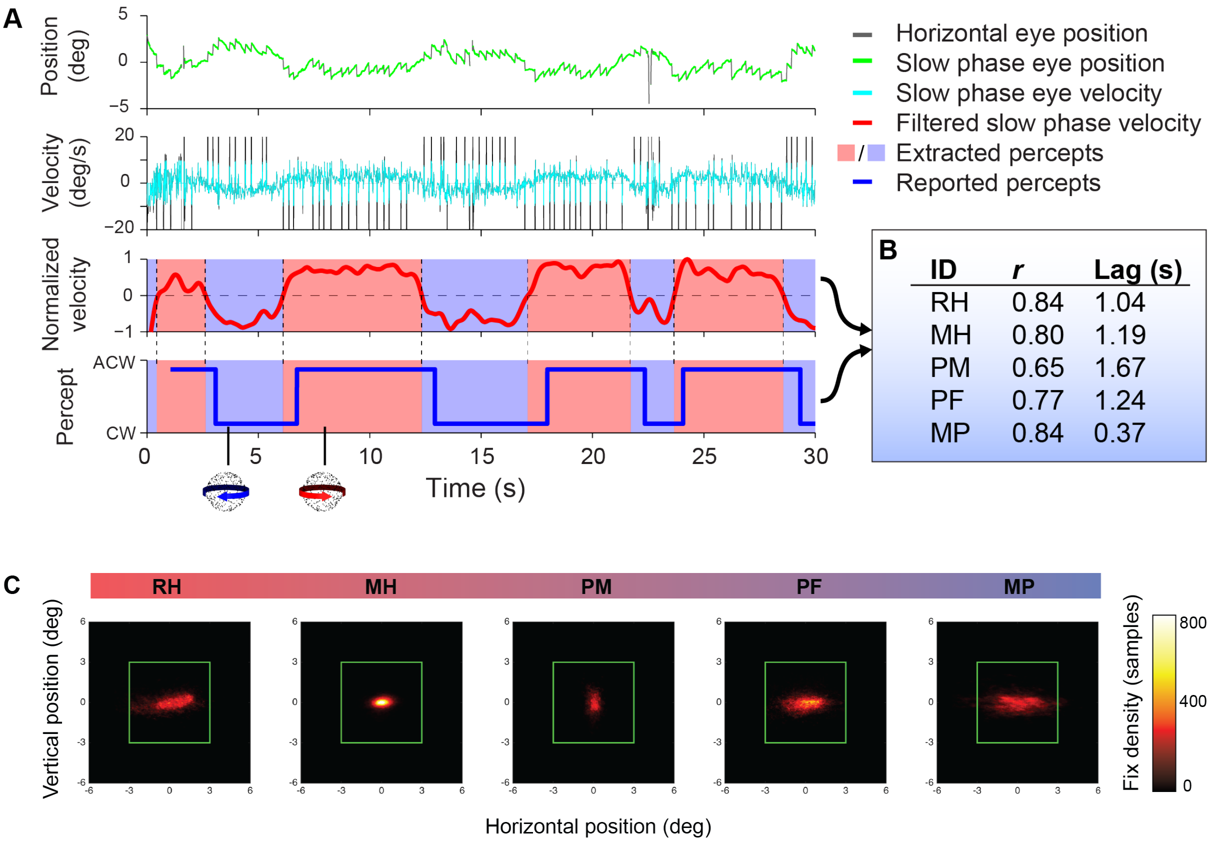

Supplementary Figure S1: A) Example data from a single trial of continuous presentation for the structure-from-motion rotating-sphere task. The upper plot shows the participant's horizontal eye position (grey trace) over time, from which a clear saw-tooth wave characteristic of optokinetic nystagmus (OKN) can be seen. The slow-phase velocities (cyan) were extracted and filtered (red), and the velocity sign (+/-) was used to infer which surface direction was perceived as the front of the rotating sphere (background colour). **B)** Cross-correlations confirmed that the perceptual time courses extracted from eye-movement data agreed closely with participants' subjective perceptual reports (blue trace in A), and alternations in OKN pattern typically preceded perceptual report of alternations by about 1 seconds on average. **C)** Distribution of gaze locations across dynamic stereo and bistable rotating sphere tasks for all five patients. The green square indicates the stimulus location on screen, while the colormap indicates fixation density (number of samples per location).

© The Authors under the terms of the Creative Commons Attribution License http://creativecommons.org/licenses/by/3.0/, which permits unrestricted use, provided the original author and source are credited.

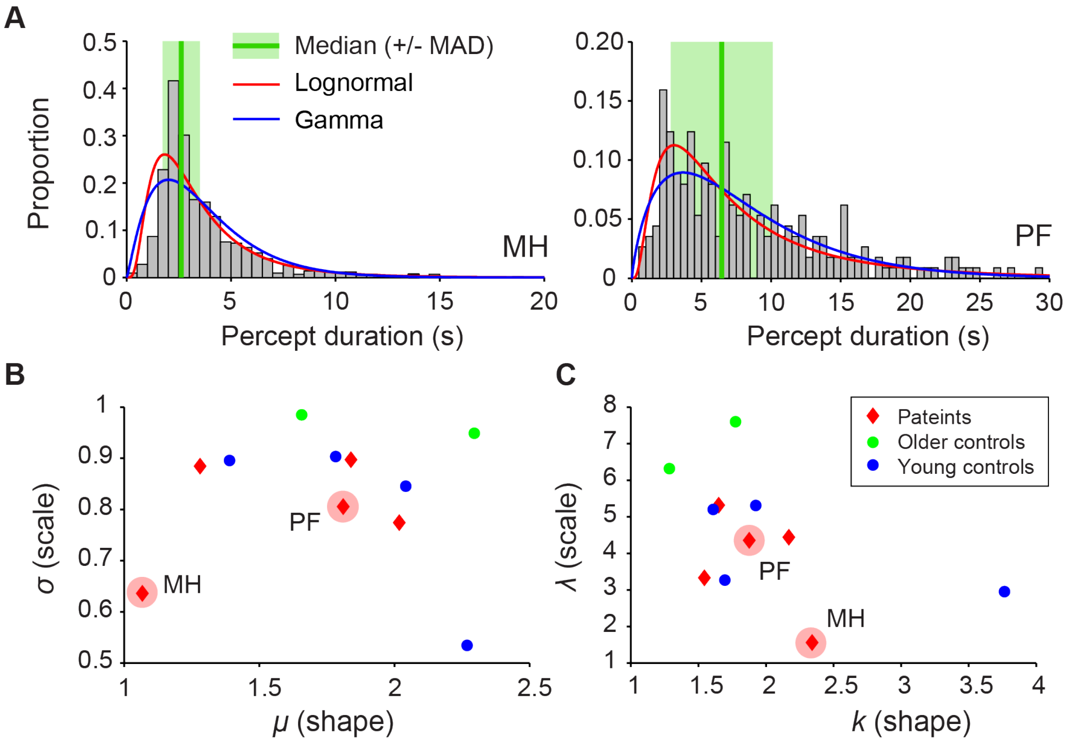

Supplementary Figure S2: Percept duration distributions for individual patients and controls on the SFM experiment. **A**) Examples of probability density fitted with lognormal (red) and gamma (blue) distributions for two patients. Vertical green lines indicate the median and median absolute deviations. **B & C**) Scatter plots showing the relationship between shape and scale parameters for the best fitting lognormal (B) and gamma (C) functions to each individual observer's percept duration distribution. The plots reveal no clear segregation of groups based on fit parameters.

© The Authors under the terms of the Creative Commons Attribution License http://creativecommons.org/licenses/by/3.0/, which permits unrestricted use, provided the original author and source are credited.

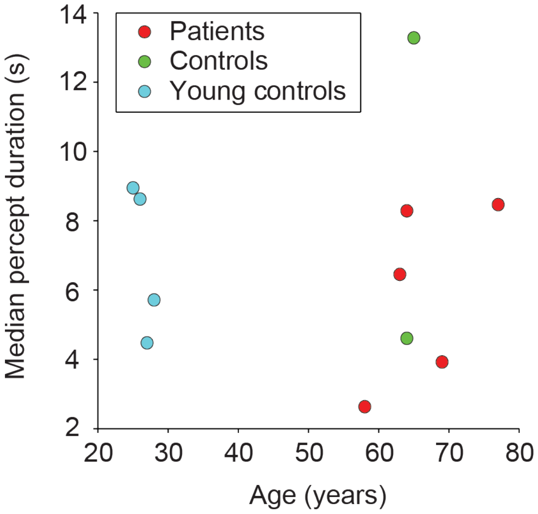

Supplementary Figure S3: Scatter plot of observer age against mean rotating sphere percept duration. While ages were bimodally distributed, there was no clear correlation between age and percept duration.

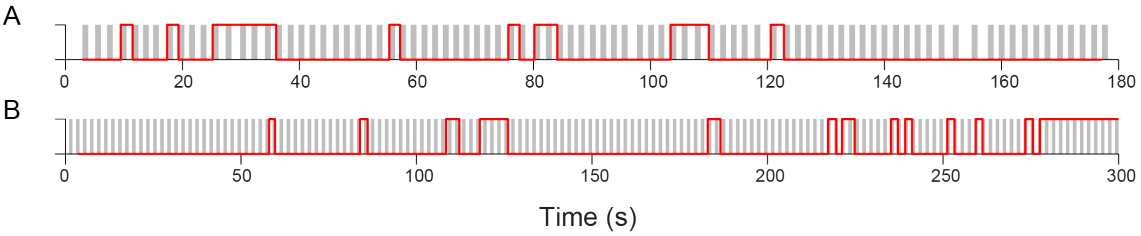

Supplementary Figure S4: A) Perceptual reports for patient PF during one block of the dynamic disparity task. On this task, inter-trial intervals were always 1 second (grey bars), but stimulus duration was self-paced (i.e. variable) and averaged 1.03 ± 0.04s. The stimulus rotated clockwise on half of the trials in each block, and this result therefore shows a strong bias. B) Perceptual reports for the same patient during intermittent presentation of the bistable rotating sphere stimulus (i.e. similar to the dynamic disparity task, but without disparity).

© The Authors under the terms of the Creative Commons Attribution License http://creativecommons.org/licenses/by/3.0/, which permits unrestricted use, provided the original author and source are credited.

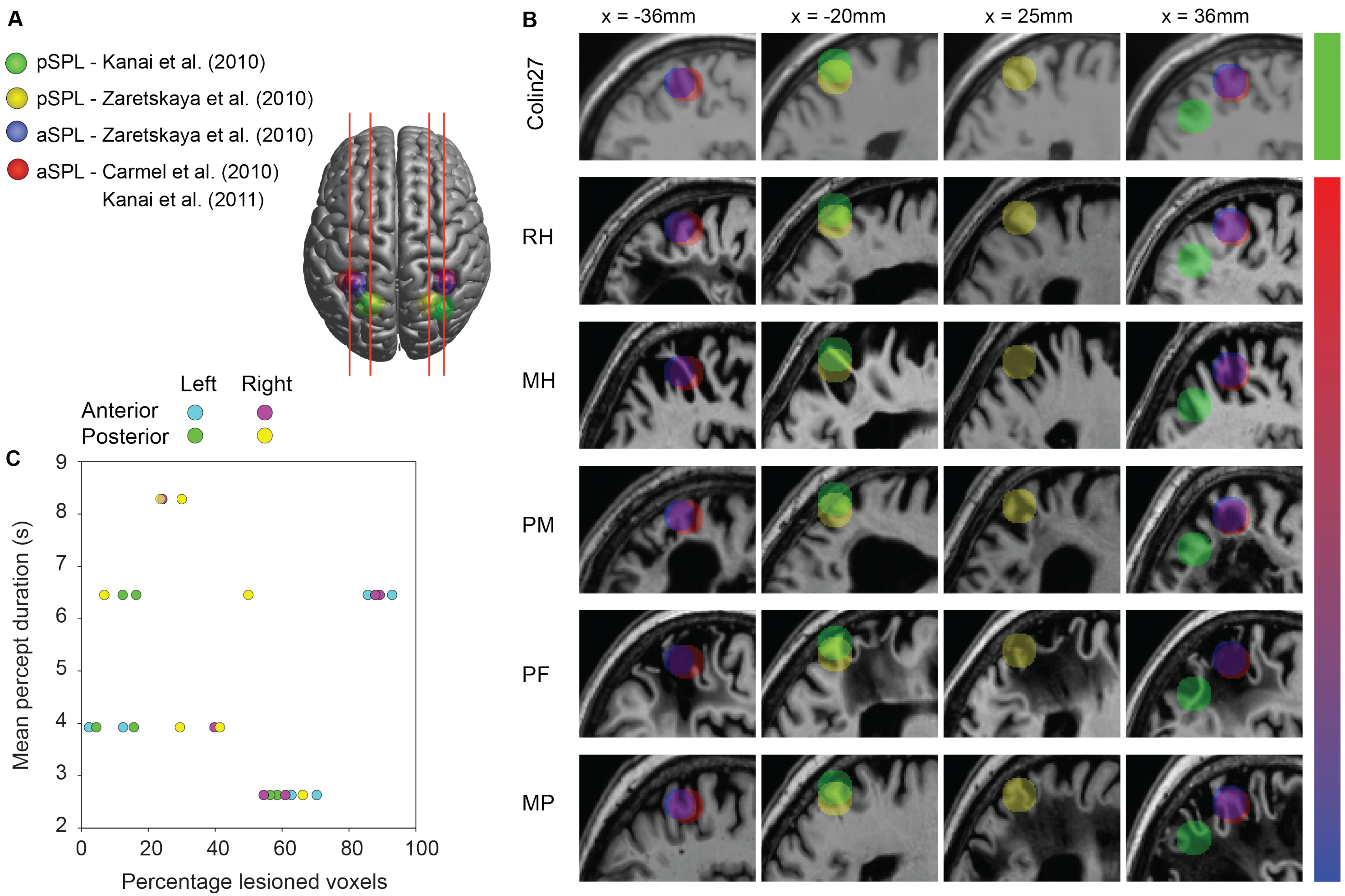

Supplementary Figure S5: Region-of-interest (ROI) analysis. A) Four ROIs were created in the PPC of each hemisphere, based on a spherical volume (10mm radius) centred on MNI coordinates reported by previous studies (**Supplementary Table 3**). B) Overlap between each of the 8 ROIs and the lesion mask of each patient was quantified. C) The mean percept duration on the bistable rotating sphere task for each patient was plotted against the proportion of lesioned voxels for each ROI (note the legend colours correspond to left and right ROIs separately).

© The Authors under the terms of the Creative Commons Attribution License http://creativecommons.org/licenses/by/3.0/, which permits unrestricted use, provided the original author and source are credited.

Supplementary Table S1: Percentage correct responses during catch periods for bistable experiments.

Subject	Structure-from-motion	Apparent motion	Binocular rivalry
RH	89% (25/28)	-	75% (3/4)
MH	98% (118/120)	70% (7/10)	60% (6/10)
PM	54% (13/24)	89% (16/18)	-
PF	80% (12/15)	100% (4/4)	-
MP	100% (4/4)	-	100% (4/4)
DC	86% (12/14)	100% (2/2)	100% (1/1)
RC	100% (4/4)	67% (2/3)	-

Supplementary Table S2: Mean reaction times (± SEM, ms) for binocular disparity tasks.

Subject	Dynamic	Fine	Signal-in-noise
RH	3.085 ± 0.092		NA
MH	1.169 ± 0.032	1.311 ± 0.029	1.378 ± 0.029
PM	2.584 ± 0.059		NA
PF	1.529 ± 0.045		NA
MP	2.275 ± 0.073		
DC	0.658 ± 0.022		NA
RC	1.260 ± 0.047		NA

© The Authors under the terms of the Creative Commons Attribution License http://creativecommons.org/licenses/by/3.0/, which permits unrestricted use, provided the original author and source are credited.

Supplementary Table S2: Individual percept duration distribution statistics for the rotating sphere stimulus. All summary statistics are given in seconds (3dp).

	Summary statistics					
**Subject**	**Mean**	**SD**	**SE**	**Median**	**32% CI**	**68% CI**
RH	9.636	6.249	0.544	8.465	5.577	11.892
MH	3.655	3.246	0.112	2.631	2.099	3.631
PM	5.146	4.818	0.318	3.924	2.474	5.476
PF	8.169	6.038	0.403	6.454	4.171	9.930
MP	8.775	7.002	1.698	8.285	4.422	9.800
DC	8.116	7.360	0.624	4.607	3.205	8.652
RC	13.502	8.354	1.638	13.277	9.698	17.928
APM	8.370	6.444	0.609	5.715	4.101	10.599
MLP	10.214	6.892	0.836	8.627	6.187	11.495
JMB	11.110	5.997	0.622	8.952	7.339	12.930
CH	5.543	4.220	0.554	4.477	3.140	6.367

Lognormal fit data

**μ (shape)**	**μ CI**	**μ CI**	**σ (scale)**	**σ CI**	**σ CI**	**df**	**N**	**χ^2**	***p***
2.018	1.884	2.151	0.774	0.691	0.881	5	132	**6.656**	0.248
1.067	1.025	1.110	0.636	0.607	0.668	3	846	35.721	0.000
1.281	1.166	1.396	0.885	0.810	0.974	3	229	2.935	0.402
1.811	1.705	1.916	0.806	0.737	0.888	5	225	**7.702**	0.173
1.839	1.378	2.301	20.897	0.668	1.366	0	17	**0.777**	-
1.657	1.492	1.822	0.985	0.881	1.117	4	139	9.958	0.041
2.296	1.912	2.679	0.949	0.744	1.310	0	26	**2.260**	-
1.783	1.614	1.952	0.903	0.799	1.040	4	112	**10.861**	0.028
2.042	1.837	2.247	0.846	0.724	1.018	3	68	**5.986**	0.112
2.269	2.159	2.379	0.535	0.467	0.625	4	93	1.011	0.908
1.390	1.154	1.625	0.896	0.757	1.097	2	58	**1.300**	0.522

Gamma fit data

**k (shape)**	**k CI**	**k CI**	**λ (scale)**	**λ CI**	**λ CI**	**df**	**N**	**χ^2**	***p***
2.169	1.732	2.717	4.442	3.448	5.722	5	132	2.256	0.813
2.341	2.141	2.560	1.561	1.413	1.725	2	846	**99.648**	0.000
1.544	1.306	1.825	3.333	2.737	4.058	3	229	**3.257**	0.354
1.876	1.581	2.225	4.355	3.581	5.297	5	225	4.319	0.504
1.650	0.891	3.056	5.318	2.591	10.912	0	17	0.191	-
1.285	1.040	1.588	6.315	4.882	8.169	5	139	**16.904**	0.005

© The Authors under the terms of the Creative Commons Attribution License http://creativecommons.org/licenses/by/3.0/, which permits unrestricted use, provided the original author and source are credited.

1.776	1.076	2.930	7.604	4.266	13.553	0	26	0.412	-
1.609	1.266	2.045	5.202	3.929	6.887	5	112	9.590	0.088
1.924	1.409	2.627	5.308	3.720	7.573	3	68	4.755	0.191
3.765	2.858	4.960	2.951	2.197	3.963	4	93	**2.426**	0.658
1.696	1.214	2.369	3.268	2.216	4.819	2	58	0.591	0.744

© The Authors under the terms of the Creative Commons Attribution License http://creativecommons.org/licenses/by/3.0/, which permits unrestricted use, provided the original author and source are credited.

Supplementary Table S3: Parietal regions causally involved in perceptual bistability. Durations show mean change in mean percept duration following TMS to the parietal target sites compared to vertex. BR = Binocular rivalry; RS = rotating sphere; MIB = motion-induced blindness; cTBS = continuous theta burst stimulation; rTMS = repetitive TMS; Tal. = Talairach coordinates. (*Ryota Kanai, personal communication, August 12, 2014).

Study	Stimulus	TMS method	Region	MNI coordinates (mm) x	y	z	Δ mean percept duration (ms)	Significance *P*-value
(21)	BR	Online TMS (2Hz)	R pSPL	24.12	-61.66	57.75	+ 110	$p = 0.064$
			L pSPL	-18.79	-62.34	55.30	+ 20	$p > 0.05$
			R aSPL	35.84	-46.39	53.69	+ 220	$p = 0.0015$
			L aSPL	-31.87	-48.28	52.87	+ 80	$p > 0.05$
(22)	BR	1Hz rTMS	R aSPL	36	-45	51	- 500	$p = 0.02$
			L aSPL	-36	-45	51	+ 220	$p > 0.05$
(6)	RS	cTBS	R pSPL*	34	-66	34	+ 1400? (+ 24%)	$p < 0.01$
			L pSPL*	-21	-63	61	+ 1100? (+ 20%)	$p < 0.01$
(20)	RS	cTBS	R aSPL	36	-45	51	- 885? (- 15%)	$p < 0.05$
(23)	RS	rTMS	R SPL	27 (Tal.)	-71 (Tal.)	42 (Tal.)	- 2%	$p = 0.83$
(24)	MIB	cTBS	R PPC	42	-58	52	+ 250	$p < 0.005$

Supplementary Table S4. Percentage of voxels within each parietal ROI classified as lesioned. *A = ROIs based on coordinates from (6, 22) (Ryota Kanai, personal communication, August 22nd 2014). B = Corresponding but non-overlapping ROIs based on coordinates from (21).

				Group A				Group B			
Patient	Stereo	Switch rate	Lesion side	Left pSPL	Right pSPL	Left aSPL	Right aSPL	Left pSPL	Right pSPL	Left aSPL	Right aSPL
RH	+	+	L	0	0	0	0	0	0	0	0
MH	+	-	L/B	70	63	55	61	59	56	0	66
PM	-	-	B	13	16	40	41	2	5	0	29
PF	-	+	R	86	93	89	88	12	16	7	50
MP	-	+	R	0	0	24	24	0	0	0	30

© The Authors under the terms of the Creative Commons Attribution License http://creativecommons.org/licenses/by/3.0/, which permits unrestricted use, provided the original author and source are credited.

References

1. Borsellino A, De Marco A, Allazetta A, Rinesi S, Bartolini B. Reversal time distribution in the perception of visual ambiguous stimuli. Kybernetik. 1972;10(3):139-44.
2. Logothetis NK, Leopold DA, Sheinberg DL. What is rivalling during binocular rivalry? Nature. 1996;380(6575):621-4.
3. Levelt WJ. Note on the distribution of dominance times in binocular rivalry. Br J Psychol. 1967;58(1):143-5.
4. Krug K, Brunskill E, Scarna A, Goodwin GM, Parker AJ. Perceptual switch rates with ambiguous structure-from-motion figures in bipolar disorder. Proceedings of the Royal Society B: Biological Sciences. 2008;275(1645):1839-48.
5. Zhou Y, Gao J, White K, Merk I, Yao K. Perceptual dominance time distributions in multistable visual perception. Biological cybernetics. 2004;90(4):256-63.
6. Kanai R, Bahrami B, Rees G. Human parietal cortex structure predicts individual differences in perceptual rivalry. Current Biology. 2010;20(18):1626-30.
7. Leopold D, Maier A, Logothetis NK. Measuring subjective visual perception in the nonhuman primate. Journal of Consciousness Studies. 2003;10(9-10):9-10.
8. Fox R, Todd S, Bettinger LA. Optokinetic nystagmus as an objective indicator of binocular rivalry. Vision research. 1975;15(7):849-53.
9. Frässle S, Sommer J, Jansen A, Naber M, Einhäuser W. Binocular rivalry: frontal activity relates to introspection and action but not to perception. The Journal of Neuroscience. 2014;34(5):1738-47.
10. Riddoch MJ, Humphreys GW, Edwards S, Baker T, Willson K. Seeing the action: neuropsychological evidence for action-based effects on object selection. Nature neuroscience. 2002;6(1):82-9.
11. Kitadono K, Humphreys GW. Interactions between perception and action programming: Evidence from visual extinction and optic ataxia. Cognitive neuropsychology. 2007;24(7):731-54.
12. Snow JC, Miranda RR, Humphreys GW. Impaired visual sensitivity within the ipsilesional hemifield following parietal lobe damage. Cortex. 2013;49(1):158-71.
13. Cavina-Pratesi C, Connolly JD, Milner AD. Optic ataxia as a model to investigate the role of the posterior parietal cortex in visually guided action: evidence from studies of patient MH. Frontiers in human neuroscience. 2013;7.
14. Rice NJ, Edwards MG, Schindler I, Punt TD, McIntosh RD, Humphreys GW, et al. Delay abolishes the obstacle avoidance deficit in unilateral optic ataxia. Neuropsychologia. 2008;46(5):1549-57.
15. Dent K, Lestou V, Humphreys GW. Deficits in visual search for conjunctions of motion and form after parietal damage but with spared hMT+/V5. Cognitive neuropsychology. 2010;27(1):72-99.
16. Dombrowe I, Donk M, Wright H, Olivers CN, Humphreys GW. The contribution of stimulus-driven and goal-driven mechanisms to feature-based selection in patients with spatial attention deficits. Cognitive neuropsychology. 2012;29(3):249-74.
17. Vancleef K, Wagemans J, Humphreys GW. Impaired texture segregation but spared contour integration following damage to right posterior parietal cortex. Experimental brain research. 2013;230(1):41-57.
18. Vivas AB, Humphreys GW, Fuentes LJ. Object-based inhibition of return in patients with posterior parietal damage. Neuropsychology. 2008;22(2):169.
19. Correani A, Humphreys GW. An impaired attentional dwell time after parietal and frontal lesions related to impaired selective attention not unilateral neglect. Cognitive neuropsychology. 2011;28(5):363-85.
20. Kanai R, Carmel D, Bahrami B, Rees G. Human parietal cortex structure determines individual differences in perceptual rivalry. Journal of Vision. 2011;11(11):299-.
21. Zaretskaya N, Thielscher A, Logothetis NK, Bartels A. Disrupting parietal function prolongs dominance durations in binocular rivalry. Current biology. 2010;20(23):2106-11.
22. Carmel D, Walsh V, Lavie N, Rees G. Right parietal TMS shortens dominance durations in binocular rivalry. Current Biology. 2010;20(18):R799-R800.
23. de Graaf TA, de Jong MC, Goebel R, van Ee R, Sack AT. On the functional relevance of frontal cortex for passive and voluntarily controlled bistable vision. Cerebral Cortex. 2011:bhr015.
24. Nuruki A, Oliver R, Campana G, Walsh V, Rothwell JC. Opposing roles of sensory and parietal cortices in awareness in a bistable motion illusion. Neuropsychologia. 2013;51(13):2479-84.

© The Authors under the terms of the Creative Commons Attribution License http://creativecommons.org/licenses/by/3.0/, which permits unrestricted use, provided the original author and source are credited.
